# The generation and analyses of a novel combination of recombinant adenovirus vaccines targeting three tumor antigens as an immunotherapeutic

**DOI:** 10.18632/oncotarget.5181

**Published:** 2015-09-07

**Authors:** Elizabeth S. Gabitzsch, Kwong Yok Tsang, Claudia Palena, Justin M. David, Massimo Fantini, Anna Kwilas, Adrian E. Rice, Yvette Latchman, James W. Hodge, James L. Gulley, Ravi A. Madan, Christopher R. Heery, Joseph P. Balint, Frank R. Jones, Jeffrey Schlom

**Affiliations:** ^1^ Etubics Corporation, Seattle, WA, USA; ^2^ Laboratory of Tumor Immunology and Biology, Center for Cancer Research, National Cancer Institute, National Institutes of Health, Bethesda, MD, USA; ^3^ Genitourinary Malignancies Branch, Center for Cancer Research, National Cancer Institute, National Institutes of Health, Bethesda, MD, USA

**Keywords:** cancer vaccines, adenovirus vaccines, tumor antigens, immunotherapy, brachyury

## Abstract

Phenotypic heterogeneity of human carcinoma lesions, including heterogeneity in expression of tumor-associated antigens (TAAs), is a well-established phenomenon. Carcinoembryonic antigen (CEA), MUC1, and brachyury are diverse TAAs, each of which is expressed on a wide range of human tumors. We have previously reported on a novel adenovirus serotype 5 (Ad5) vector gene delivery platform (Ad5 [E1-, E2b-]) in which regions of the early 1 (E1), early 2 (E2b), and early 3 (E3) genes have been deleted. The unique deletions in this platform result in a dramatic decrease in late gene expression, leading to a marked reduction in host immune response to the vector. Ad5 [E1-, E2b-]-CEA vaccine (ETBX-011) has been employed in clinical studies as an active vaccine to induce immune responses to CEA in metastatic colorectal cancer patients. We report here the development of novel recombinant Ad5 [E1-, E2b-]-brachyury and-MUC1 vaccine constructs, each capable of activating antigen-specific human T cells *in vitro* and inducing antigen-specific CD4^+^ and CD8^+^ T cells in vaccinated mice. We also describe the use of a combination of the three vaccines (designated Tri-Ad5) of Ad5 [E1-, E2b-]-CEA, Ad5 [E1-, E2b-]-brachyury and Ad5 [E1-, E2b-]-MUC1, and demonstrate that there is minimal to no “antigenic competition” in *in vitro* studies of human dendritic cells, or in murine vaccination studies. The studies reported herein support the rationale for the application of Tri-Ad5 as a therapeutic modality to induce immune responses to a diverse range of human TAAs for potential clinical studies.

## INTRODUCTION

An advanced viral vector gene delivery platform based upon a recombinant adenovirus serotype-5 (Ad5), referred to as Ad5 [E1-, E2b-], can be utilized as a vaccine and immunotherapy modality even in the presence of pre-existing immunity against adenovirus [1–10]. The platform consists of a replication defective Ad5 in which portions of the early 1 (E1), early 2 (E2b) and early 3 (E3) Ad5 gene regions have been deleted [11, 12]. The deletions in the E2b region, specifically the DNA polymerase and preterminal protein, have been reported to result in a dramatic decrease of late gene expression, such as Ad5 fiber [12], which results in a marked reduction in host inflammatory responses to the vector and the associated toxicity [13]. Human cells transfected with these Ad5 [E1-, E2b-] constructs were shown to express the encoded transgene(s) for prolonged times *in vivo* compared to other vector platforms [4, 5], as the lack of Ad5 late gene expression in the proprietary platform renders infected antigen-presenting cells (APCs) less vulnerable to anti-Ad5 immunity, and permits them to produce and express inserted transgenes for extended periods of time [14]. Administration of these vaccines resulted in specific immunization and immunotherapy against infectious diseases and cancers [1–10]. In a Phase I/II clinical trial, cohorts of patients with metastatic colorectal cancer (mCRC) were vaccinated with escalating doses of the Ad5 [E1-, E2b-] platform carrying a gene for carcinoembryonic antigen (CEA) [1, 10]. CEA represents an attractive target for immunotherapy since it is overexpressed in the majority of human carcinomas [15, 16]. Ad5 [E1-, E2b-]-CEA was well tolerated in mCRC patients and CEA-directed T-cell responses were induced in a dose-responsive manner [10]; no significant changes in Treg:Teffector cell ratios were noted in this trial [1]. Patients in this study exhibited evidence of a favorable survival probability, with all 25 patients treated at least two times with Ad5 [E1-, E2b-]-CEA exhibiting a 12-month overall survival probability of 48%, with a mean overall survival of 11 months [1, 10].

The phenotypic heterogeneity in terms of expression of different tumor-associated antigens (TAAs) in a given primary or metastatic tumor mass is a well-established phenomenon [17–21]. One can speculate that the use of an immunotherapeutic vaccine regimen targeting three distinct TAAs, each of which is widely expressed on the majority of human carcinomas, would be potentially therapeutically advantageous over the use of a vaccine targeting only one TAA. With the safety and immunogenicity of Ad5 [E1-, E2b-]-CEA established in patients as a single agent, we now investigate a multi-target approach. We previously reported that a human immunodeficiency virus (HIV) vaccine containing four adenovirus constructs expressing Gag, Pol, Nef or Env could elicit an immune response to all four antigens when given simultaneously, even in the presence of Ad5 immunity [3].

Brachyury is a member of the T-box family of transcription factors that play key roles during early development, mostly in the formation and differentiation of normal mesoderm, which is characterized by a highly conserved DNA binding domain designated as the T-box [22]. Recently, the epithelial-mesenchymal transition (EMT) has been recognized as a key step during the progression of primary tumors into a metastatic state, in which brachyury plays a crucial role [23–25]. Brachyury expression is undetectable or minimally expressed in most normal adult human tissues and is overexpressed in multiple human cancers [24]. In addition, expression of brachyury has been shown to be associated with poor prognosis of colorectal [26], lung [27], prostate [28], hepatocellular [29], and breast [30] carcinomas. Brachyury overexpression in human tumor cells has also been associated with drug resistance [31, 32]. Transcription factors have been considered “difficult to drug” due to their primary location in the nucleus and lack of a hydrophobic groove for drug attachment. Studies have shown, however, that brachyury-specific T cells can be generated both *in vitro* and *in vivo* and that these T cells have the ability to lyse human tumors endogenously expressing brachyury [22, 33, 34]. Patients generate brachyury-specific T cells post-vaccination using vaccines expressing CEA and prostate-specific antigen (PSA), indicating the potential immunogenicity of brachyury in humans [35, 36]. A recently completed Phase I study [37] with a recombinant *Saccharomyces cerevisiae* brachyury vaccine also revealed the generation of brachyury-specific T cells, thus providing further evidence of immunogenicity.

MUC1 (CD227) is a TAA that is overexpressed on a majority of human carcinomas and several hematologic malignancies [38–41]. MUC1 is normally expressed at the surface of glandular epithelial cells [42] and, in carcinomas, it is overexpressed and aberrantly hypoglycosylated [38, 42, 43]. Several clinical trials have been and are being performed to evaluate the use of MUC1 in immunotherapeutic vaccines [44–48]. Some of these trials have indicated that targeting MUC1 is safe and may provide survival benefit [45, 47, 49]. We have previously identified multiple enhancer agonist epitopes, several of which are in the MUC1 C-terminus region [50, 51]. This is potentially important because numerous studies [52–56] have demonstrated that the C-terminus of MUC1 has oncogenic potential, associates with poor prognosis and drug resistance, and induces “stemness” features in a range of human carcinomas. The human T-cell lines generated using these MUC1 agonist epitopes were more efficient than those generated with the corresponding native epitopes in terms of antigen-specific interferon (IFN)–γ production and lysis of tumor cells endogenously expressing native MUC1 [50, 51]. Therefore, we believe that MUC1 containing modified agonist epitopes has a greater potential as an immunogenic agent for vaccine development.

The Ad5 [E1-, E2b-]-CEA vector, which encoded the entire CEA sequence modified to express an enhancer T-cell epitope, has been described previously [1, 6, 10]. Studies were undertaken to determine whether recombinant Ad5 [E1-, E2b-]-MUC1 and Ad5 [E1-, E2b-]-brachyury constructs could be developed that have the ability to generate MUC1- and brachyury-specific T-cell responses. Ad5 [E1-, E2b-]-brachyury was constructed to encode the entire brachyury gene devoid of 25 amino acids involved in DNA binding and modified to express an enhancer T-cell epitope; Ad5 [E1-, E2b-]-MUC1 was constructed to encode the entire MUC1 transgene with eight of the agonist epitopes previously described above [50, 51], including those in the C-terminus region. One potential pitfall in the use of an admixture of three vectors, each containing a different TAA, is that there may be “antigenic competition” and one or two TAAs would become dominant as to greatly inhibit or eliminate the expression of another TAA. Studies were thus designed to determine whether a mixture of Ad5 [E1-, E2b-]-CEA, Ad5 [E1-, E2b-]-MUC1, and Ad5 [E1-, E2b-]-brachyury (designated Tri-Ad5) has the ability to generate human T-cell responses *in vitro*, and murine T-cell responses *in vivo*, to each of the TAA transgenes.

## RESULTS

**Figure 1:**
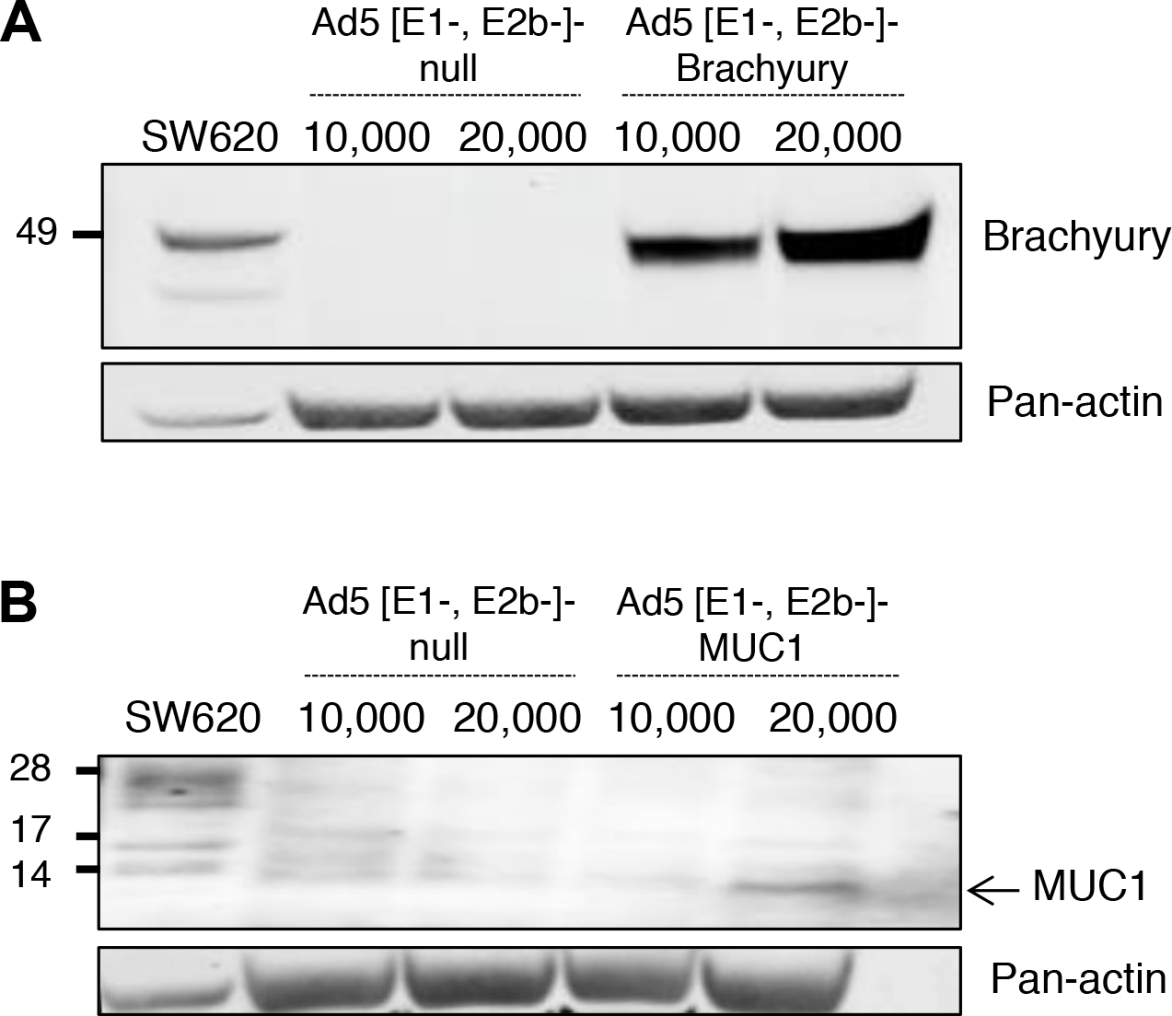
Expression of brachyury and MUC1 protein in human dendritic cells (DCs) infected with Ad5 [E1-, E2b-]- brachyury and Ad5 [E1-, E2b-]-MUC1 SW620 tumor cells were used as positive control. Actin was used as a loading control. **A.** Expression of brachyury was robust in DCs infected with Ad5 [E1-, E2b-]-brachyury. **B.** MUC1 expression was observed in human DCs infected with Ad5 [E1-, E2b-]-MUC1 vector as compared to DCs infected with Ad5 [E1-, E2b-]-null (no transgene).

Recombinant Ad5 [E1-, E2b-]-CEA was generated and characterized as previously described [6]. Recombinant Ad5 [E1-, E2b-]-MUC1 and Ad5 [E1-, E2b-]-brachyury were generated as described in the Methods section. As seen in Figure 1A, Western blot analysis using an anti-brachyury–specific monoclonal antibody (MAb 54–1) [57] revealed brachyury expression when human dendritic cells (DCs) were infected with Ad5 [E1-, E2b-]-brachyury. An Ad5 [E1-, E2b-] vector devoid of any transgene (Ad5 [E1-, E2b-]-null) was used as a negative control and SW620 human colon carcinoma cells that endogenously express brachyury were used as a positive control. An anti-MUC1–specific MAb was used to detect the expression of MUC1 in Ad5 [E1-, E2b-]-MUC1–infected human DCs (Figure 1B). SW620 cells, which also express MUC1 endogenously, were used as a positive control. The difference in molecular weights seen in the human DCs versus the SW620 human carcinoma cells is most likely due to the differential glycosylation of the MUC1 protein. As has been previously shown by others [58–61], it would appear that MUC1-C is being expressed in the human DCs predominantly as the unglycosylated 17 or 15 kDa form and not the 25–20 glycosylated species. A Western blot of Ad5 [E1-, E2b-]-CEA–infected human cells is shown in Supplemental Figure 1. Human DCs infected with Ad5 [E1-, E2b-]-CEA, Ad5 [E1-, E2b-]-MUC1 and Ad5 [E1-, E2b-]-null were analyzed for evidence of DC maturation versus uninfected human DCs. There were no differences between the Ad5 [E1-, E2b-]-null and the recombinant Ad5 [E1-, E2b-] vectors expressing the TAAs in that each slightly upregulated surface CD80 and CD83 expression and strongly upregulated HLA-DR surface expression (Supplemental Table 1); it is thus apparent that any changes in DC maturation are due to the Ad5 vector alone and not any TAA transgene insertion.

We have previously reported on the generation of brachyury-, CEA-, and MUC1-specific human CD8^+^ T cells employing the corresponding peptide for each TAA [22, 50, 51, 62–64]. As shown in Table 1, Ad5 [E1-, E2b-]-null did not activate any of the T cells to produce IFN-γ. Ad5 [E1-, E2b-]-brachyury–infected DCs activated brachyury-specific T cells and not CEA-specific T cells (as a negative control). This demonstrates that the Ad5 [E1-, E2b-]-brachyury–infected DCs could process brachyury in a manner that generates brachyury–MHC Class I complexes capable of specific T-cell activation. Similarly, Ad5 [E1-, E2b-]-CEA–infected DCs specifically activated CEA-specific T cells but not MUC1-specific T-cell lines. Both Class I HLA-A2 and -A24 MUC1-specific T-cell lines have been previously generated [51] and the Ad5 [E1-, E2b-]-MUC1–infected DCs were capable of activating both of these T-cell lines but not the CEA-specific T-cell line (Table 1A). Human DCs were similarly infected with the Tri-Ad5 vector. As seen in Table 1B, T cells specific for CEA, MUC1, and brachyury were each activated to induce similar levels of IFN-γ as seen with the use of the individual Ad5 vectors.

**Table 1A:** Infection of human dendritic cells with recombinant adenovirus vectors encoding CEA, MUC1 or brachyury can activate antigen-specific T-cell lines

Dendritic cells (DCs) infected with	Antigen-specific T-cell lines			
	CEA	MUC1(HLA-A2)	MUC1(HLA-A24)	Brachyury
Ad5 [E1-, E2b-]-null	<15.6	<15.6	<15.6	<15.6
Ad5 [E1-, E2b-]-brachyury	<15.6	—	—	**351.9**
Ad5 [E1-, E2b-]-MUC1	<15.6	**335.2**	**806.4**	—
Ad5 [E1-, E2b-]-CEA	**350.0**	<15.6	<15.6	—
Uninfected DCs	<15.6	<15.6	<15.6	<15.6
T cells only	<15.6	<15.6	<15.6	<15.6

Human DCs (6-day culture in IL-4 and granulocyte-macrophage colony-stimulating factor (GM-CSF) 2 × 10^4^ cells/well in 0.5 ml of AIM-V) were infected with indicated adenovirus vectors at 20,000 multiplicity of infection (MOI). After 48 hours, DCs were washed and used for stimulation of human antigen-specific T cells. Results are expressed in pg/ml of IFN-γ per 1 × 10^5^ T cells/ml. Numbers in bold indicate a significant enhancement of IFN-γ secretion compared to corresponding wells with uninfected DCs. [— indicates that the assay was not performed.]

**Table 1B:** Infection of human dendritic cells with Tri-Ad5 vectors encoding transgenes can activate antigen-specific T-cell lines to produce IFN-γ

Dendritic cells (DCs) infected with	Antigen-specific T-cell lines			
	CEA(HLA-A2)	MUC1(HLA-A2)	MUC1(HLA-A24)	Brachyury(HLA-A2)
Tri-Ad5	**480**	**236**	**763**	**496**
Ad5 [E1, E2b]–null	<15.6	<15.6	<15.6	<15.6
Uninfected DCs	<15.6	<15.6	<15.6	<15.6
T cells only	<15.6	<15.6	<15.6	<15.6

Human DCs (6-day culture in IL-4 and GM-CSF) from an HLA-A2 and -A24 donor were infected with Tri-Ad5 vector at 2 × 10^4^ /well (24-well plate) in 0.5 ml of AIM-V. Tri-Ad5 vectors were used at 20,000 MOI for 1 hour and then 1.5 ml of AIM-V were added to each well. Infected DCs were incubated for 48 hours and then washed and used for stimulation of human antigen-specific T cells. Results are expressed in pg of IFN-γ per 1 × 10^5^ T cells/ml. Numbers in bold indicate a significant enhancement of IFN-γ secretion compared to corresponding wells with uninfected DCs.

Studies were then undertaken to determine whether simultaneous infection of human DCs with the CEA/MUC1/brachyury mixture of Tri-Ad5 could generate T-cell lines specific for all three TAAs. As seen in Table 2, when the T cells were activated by incubation with autologous B cells pulsed with the corresponding peptide, and not a control peptide, specific T-cell activation was observed. For example, the brachyury-specific T-cell line, generated by infecting human DCs with Tri-Ad5, was stimulated to produce IFN-γ when incubated with autologous DCs pulsed with brachyury peptide, but was not activated with the same autologous DCs pulsed with a CEA peptide. Similar results were seen with CEA and MUC1 T-cell lines generated with Tri-Ad5–infected DCs. These results indicate the lack of so-called “antigenic competition” in the *in vitro* use of Tri-Ad5.

**Table 2:** Infection of human dendritic cells with Tri-Ad5 can generate antigen-specific T cells to brachyury, MUC1 and CEA and produce IFN-γ when stimulated with autologous B cells pulsed with the corresponding peptides

Antigen-specific T-cell lines	Peptides (10 μg/ml)			
	CEA	MUC1 (A2)	MUC1 (A24)	Brachyury
T-brachyury	<15.6	—	—	243
T-MUC1 (A2)	<15.6	174	—	—
T-MUC1 (A24)	<15.6	—	206	—
T-CEA	211	<15.6	—	—

Human dendritic cells (DCs) from a prostate cancer patient (6-day culture in IL-4 and granulocyte-macrophage colony-stimulating factor (GM-CSF) 2 × 10^4^ cells/well in 0.5 ml of AIM-V) were infected with Tri-Ad5 at 20,000 MOI. After 48 hours, infected DCs were washed and used to generate specific cytotoxic T lymphocytes (CTLs) using autologous peripheral blood mononuclear cells (PBMCs) as effectors. Following 3 cycles of *in vitro* stimulations, autologous peptide-pulsed B cells were used as antigen-presenting cells. Results are expressed in pg/ml of IFN-γ. [— indicates that the assay was not performed.]

We then investigated whether brachyury-, MUC1-, and CEA-specific human T cells generated using DCs infected with Tri-Ad5 could lyse human carcinoma cells that endogenously express these TAAs. SW620 human colon carcinoma cells express all three TAAs and possess the HLA-A2 and -A24 Class I alleles. ASPC-1 human pancreatic carcinoma cells were used as a negative control since they express the three TAAs but in the context of HLA-A1 and -A26 molecules. The results (Table 3) demonstrated that Tri-Ad5–infected human DCs can generate T cells capable of lysing, in an MHC-restricted manner, human tumor cells that endogenously express brachyury, CEA, and MUC1.

**Table 3:** Infection of human DCs with Tri-Ad5 can generate brachyury-, MUC1- and CEA-specific CTLs that efficiently lyse tumor cells expressing all three antigens

Antigen-specific T-cell lines	SW620 Brachyury^+^ MUC1^+^ CEA^+^ (HLA-A2^+^/A24^+^)	ASPC-1 Brachyury^+^ MUC1^+^ CEA^+^ (HLA-A1^+^/A26^+^)
T-brachyury	64.4 (3.6)	8.3 (2.7)
T-MUC1 (P93L)	28.5 (1.3)	2.0 (1.6)
T-MUC1 (C6A)	49.3 (3.3)	5.0 (1.8)
T-CEA	42.4 (3.7)	4.3 (1.9)

Human dendritic cells (DCs) were infected with Tri-Ad5 at 20, 000 MOI. Infected DCs were used to generate specific cytotoxic T lymphocytes (CTLs) using autologous peripheral blood monoclonal cells (PBMCs). Autologous DCs were used as antigen-presenting cells for three *in vitro* stimulations (IVS). Autologous peptide-pulsed B cells were used to re-stimulate antigen-specific CTLs for two additional IVS. The effector-to-target ratio used was 30:1; CTLs were used at IVS 5. Results are expressed in % specific lysis (SD).

**Figure 2:**
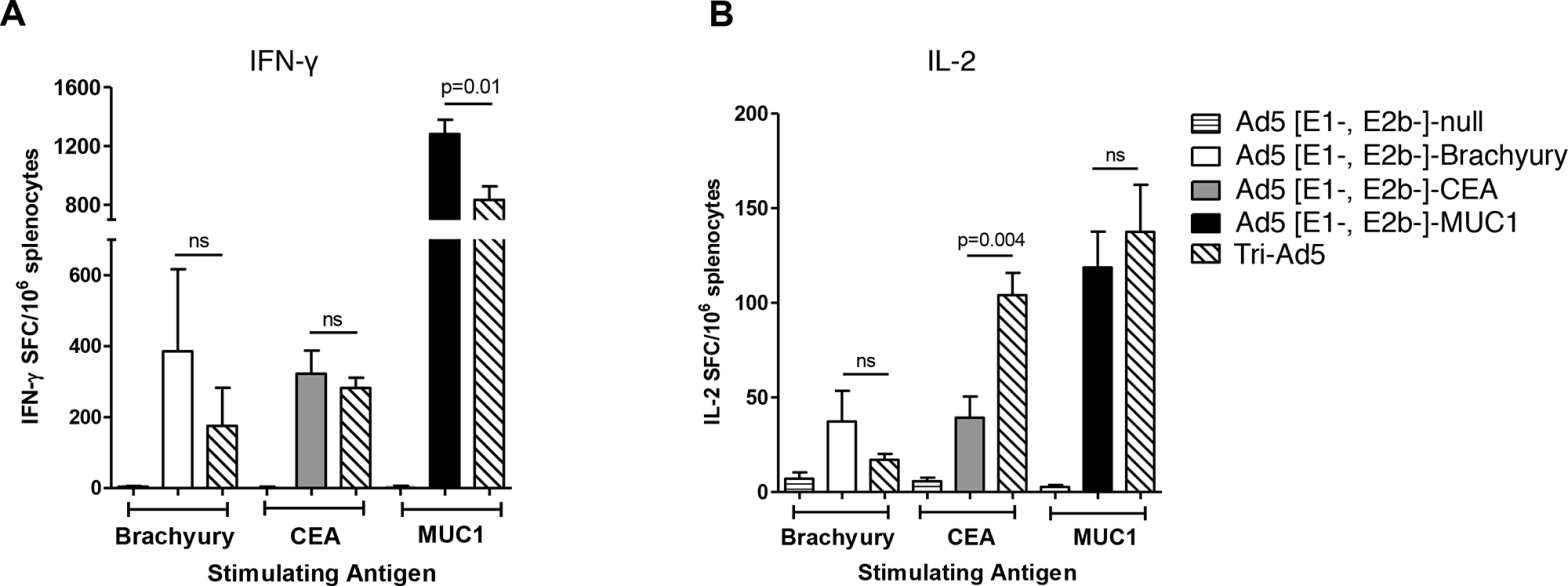
Analysis of IFN-γ− and IL-2−expressing splenocytes following vaccination of mice with Ad5 [E1-, E2b-]- brachyury, Ad5 [E1-, E2b-]-CEA, Ad5 [E1-, E2b-]-MUC1, Tri-Ad5, or Ad5 [E1-, E2b-]-null C57Bl/6 mice (*n* = 5/group) were vaccinated three times at 2-week intervals with 10^10^ VP (viral particle) of Ad5 [E1-, E2b-]-brachyury (white bar), Ad5 [E1-, E2b-]-CEA (grey bar), Ad5 [E1-, E2b-]-MUC1 (black bar) or Tri-Ad5 (1:1:1 mixture of 10^10^ VP each of Ad5 [E1-, E2b-]-brachyury, Ad5 [E1-, E2b-]-CEA, Ad5 [E1-, E2b-]-MUC1) (diagonal hatched bar). Controls received 3 × 10^10^ VP of Ad5 [E1-, E2b-]-null (horizontal striped bar). Splenocytes were collected 14 days after the final vaccination and assessed for IFN-γ−secreting cells **A.** or IL-2-secreting cells **B.** by ELISPOT assay. For positive controls, splenocytes were exposed to Concanavalin A (Con A) (data not shown). Data reported as the number of spot forming cells (SFCs) per 10^6^ splenocytes. The error bars depict the SEM. Significant differences (*p* < 0.05) between columns are reported in *p*-values, not significant = ns.

Studies were next undertaken to determine whether Ad5 [E1-, E2b-]-brachyury, Ad5 [E1-, E2b-]-MUC1, and Ad5 [E1-, E2b-]-CEA could each generate TAA-specific T-cell responses *in vivo*, and whether the Tri-Ad5 mixture could generate comparable responses. C57Bl/6 mice (*n* = 5 per group) were injected subcutaneously (s.c.) three times at 2-week intervals with 10^10^ viral particles (VP) of Ad5 [E1-, E2b-]-CEA, Ad5 [E1-, E2b-]-MUC1, Ad5 [E1-, E2b-]-brachyury, or Tri-Ad5 (1:1:1 mixture of 10^10^ VP each). An additional group of mice (*n* = 5) received 3 × 10^10^ VP of Ad5 [E1-, E2b-]-null (an empty vector control). Two weeks after the final vaccination, splenocytes from vaccinated mice were stimulated with corresponding brachyury, CEA, or MUC1 peptide pools and analyzed for IFN-γ and IL-2 secreting cells by the enzyme-linked immunospot (ELISPOT) assay. Mice vaccinated with singular constructs or with Tri-Ad5 responded to brachyury, CEA, and MUC1 peptides, respectively, with significant increases in IFN-γ and IL-2 spot forming cells (SFCs) as compared to control mice (Figure 2A and 2B). There was no significant difference in the average number of IFN-γ SFCs in mice vaccinated with Ad5 [E1-, E2b-]-brachyury or Ad5 [E1-, E2b-]-CEA individually as compared with the Tri-Ad5 vaccine. There was a significant decrease in IFN-γ SFCs in mice treated with the Tri-Ad5 vaccine as compared to Ad5 [E1-, E2b-]-MUC1 alone, although the MUC1–specific immune response induced by Tri-Ad5 remained significantly elevated over control mice (*p* < 0.0001) (Figure 2A). IL-2 responses were similar in mice treated with Tri-Ad5 versus single vaccine constructs; moreover, there was a significant increase (*p* = 0.004) in CEA-specific IL-2 SFCs when mice were vaccinated with the Tri-Ad5 vaccine versus the Ad5 [E1-, E2b-]-CEA vaccine alone (Figure 2B). Splenocytes from mice vaccinated with empty vector did not respond to brachyury, CEA, or MUC1 peptide pools. In addition, there was no reactivity to control peptide pools (simian immunodeficiency virus (SIV)–Nef and SIV-Vif) in splenocytes from any of the vaccinated groups (data not shown). Taken together, these data indicate that combining Ad5 [E1-, E2b-]-brachyury, Ad5 [E1-, E2b-]-CEA, and Ad5 [E1-, E2b-]-MUC1 in a Tri-Ad5 vaccine admixture has the effect of generating antigen-specific IFN-γ– and IL-2–producing cells similar to that achieved when using each vaccine alone.

**Figure 3:**
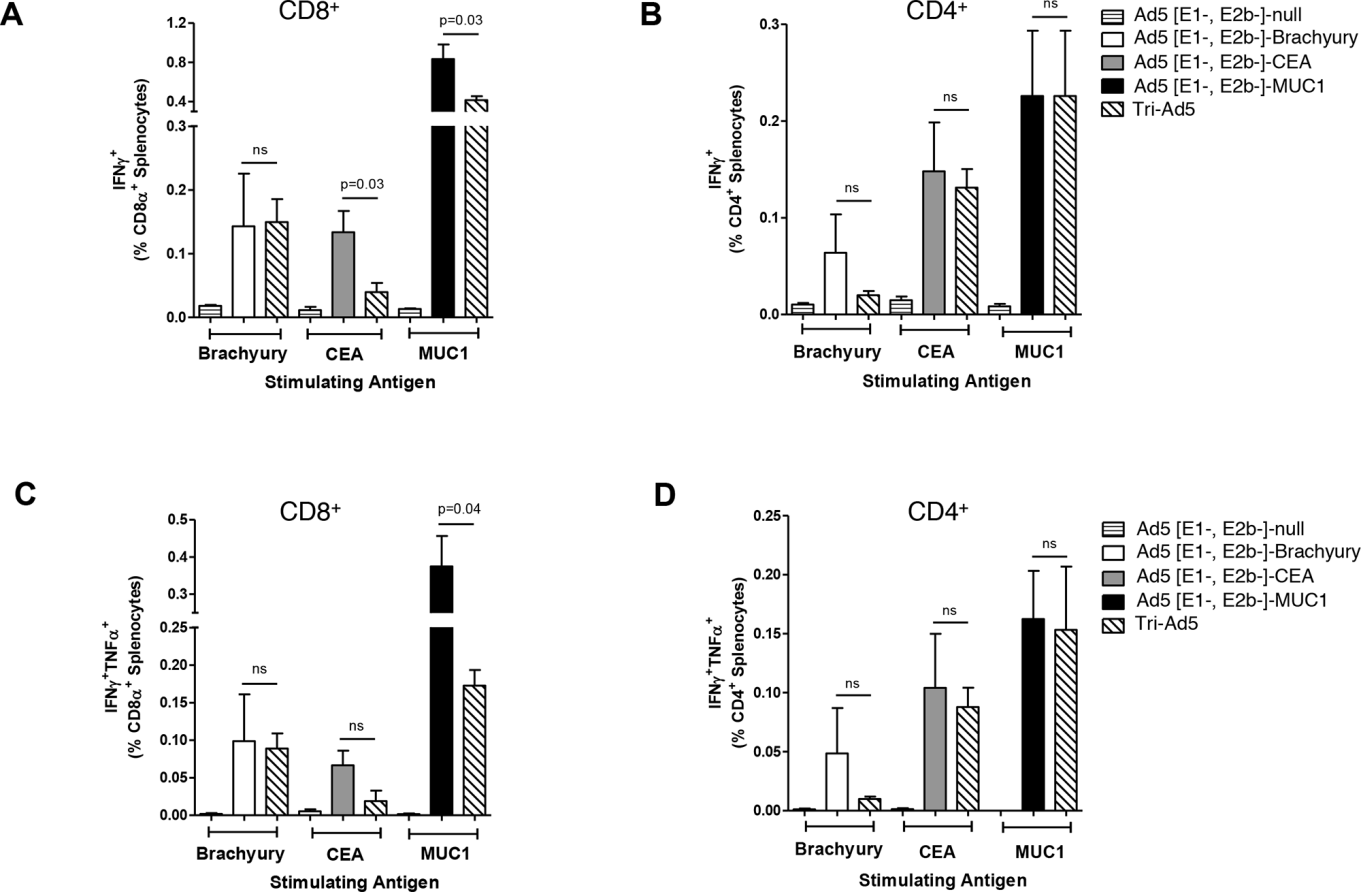
Analysis of CD8+ and CD4+ and multifunctional cellular populations following vaccination with Ad5 [E1-, E2b-]-brachyury, Ad5 [E1-, E2b-]-CEA, Ad5 [E1-, E2b-]-MUC1, Tri-Ad5, or Ad5 [E1-, E2b-]-null C57Bl/6 mice (*n* = 5/group) were vaccinated three times at 2-week intervals with 10^10^ VP (viral particle) of Ad5 [E1-, E2b-]-brachyury (white bar), Ad5 [E1-, E2b-]-CEA (grey bar), Ad5 [E1-, E2b-]-MUC1 (black bar) or Tri-Ad5 (1:1:1 mixture of 10^10^ VP (viral particle) each of Ad5 [E1-, E2b-]-brachyury, Ad5 [E1-, E2b-]-CEA, Ad5 [E1-, E2b-]-MUC1) (diagonal hatched bar). Controls received 3 × 10^10^ VP of Ad5 [E1-, E2b-]-null (horizontal striped bar). Splenocytes were collected 14 days after the final vaccination and were assessed by FACS for CD8α^+^ **A.** and CD4^+^ **B.** IFN-γ–secreting cells, or for CD8α^+^ **C.** and CD4^+^ **D.** cells secreting IFN-γ and TNF-α. For positive controls, splenocytes were exposed to Concanavalin A (Con A) (data not shown). The error bars depict the SEM. Significant differences (*p* < 0.05) between columns are reported in *p*-values, not significant = ns.

Intracellular accumulation of IFN-γ and TNF-α in CD8^+^ and CD4^+^ lymphocyte populations was also evaluated by flow cytometry using splenocytes from mice vaccinated with the adenovirus vectors and stimulated with overlapping pools of the respective synthetic peptides (Figure 3). No significant differences were observed between the IFN-γ production observed with CD8^+^ splenic lymphocytes isolated from mice vaccinated with Ad5 [E1-, E2b-]-brachyury compared with those isolated from mice vaccinated with Tri-Ad5 (Figure 3A). We did observe significant reductions between the CEA-specific and MUC1-specific IFN-γ accumulation in CD8^+^ splenocytes isolated from mice vaccinated with Tri-Ad5 as compared to single construct vaccinated mice, although the relative number of SFCs remained significantly elevated over controls (*p* < 0.0001) (Figure 3A). However, we found no significant differences in IFN-γ accumulation between CD4^+^ splenocytes isolated from each single construct vaccinated mice or Tri-Ad5 vaccinated mice (Figure 3B).

Peptide-stimulated splenocytes were also assessed by flow cytometry for the intracellular accumulation of both IFN-γ and TNF-α. We detected antigen-specific multifunctional CD8^+^ and CD4^+^ splenocytes in mice vaccinated with each single-antigen vector as well as with Tri-Ad5. When directly comparing the frequencies of dual-functional CD8^+^ and CD4^+^ splenocytes isolated from mice vaccinated with a single vector versus those from a mouse vaccinated with Tri-Ad5, we found very few differences (Figure 3C and 3D). We detected no significant differences between the dual-functional CD8^+^ splenocytes isolated from mice vaccinated with Ad5 [E1-, E2b-]-brachyury or Ad5 [E1-, E2b-]-CEA against the respective antigen as compared with those isolated from mice vaccinated with Tri-Ad5 (Figure 3C). We did observe a significant reduction in dual-functional CD8^+^ splenocytes from mice vaccinated with Ad5 [E1-, E2b-]-MUC1 compared with Tri-Ad5 (*p* = 0.04); this reduced frequency, however, was significantly elevated as compared to controls (*p* < 0.001). We found no significant differences in the frequencies of multifunctional CD4^+^ splenocytes isolated from each single construct or Tri-Ad5 vaccinated mice (Figure 3C).

**Figure 4:**
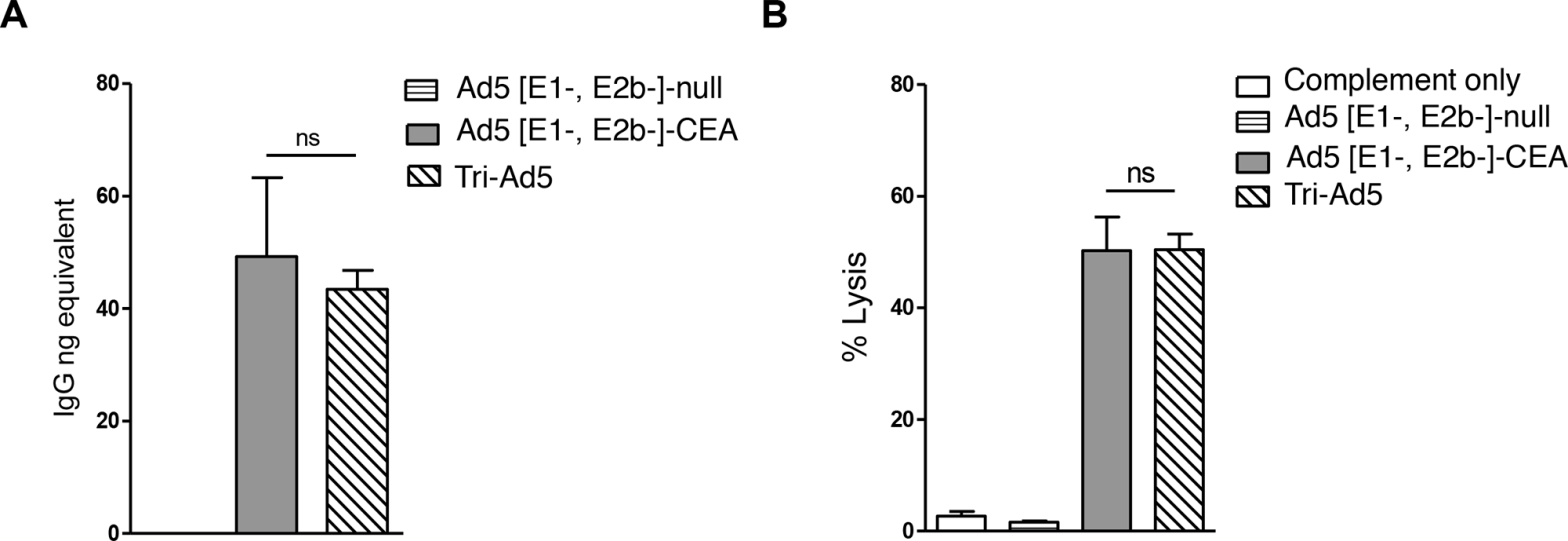
CEA antibody activity from sera from mice vaccinated with Ad5 [E1-, E2b-]-CEA or Tri-Ad5 CEA IgG levels in mice vaccinated three times with 10^10^ VP (viral particle) of Ad5 [E1-, E2b-]-CEA (grey bar), Tri-Ad5 (diagonal hatched bar) or 3 × 10^10^ VP of Ad5 [E1-, E2b-]-null (horizontal striped bar) were determined by ELISA **A.** Complement-dependent cytotoxicity (CDC) against MC38-CEA2 cells was performed **B.** The error bars depict the SEM. Significant differences (*p* < 0.05) between columns are reported in *p*-values, not significant = ns.

To assess whether humoral responses were induced by Ad5 [E1-, E2b-]-CEA, Ad5 [E1-, E2b-]-MUC1, Ad5 [E1-, E2b-]-brachyury, or Tri-Ad5 vaccines, antigen-specific quantitative enzyme-linked immunosorbent assays (ELISAs) were employed. Significant and comparable antibody responses were detected against CEA in sera from mice vaccinated with Ad5 [E1-, E2b-]-CEA or Tri-Ad5 (Figure 4A). Antibodies against CEA were not detected in mice vaccinated with control vector (Figure 4A), or mice vaccinated with Ad5 [E1-, E2b-]-brachyury, or Ad5 [E1-, E2b-]-MUC1 (data not shown). Antigen-specific antibodies to brachyury or MUC1 were not detected in sera of mice vaccinated with Ad5 [E1-, E2b-]-brachyury, Ad5 [E1-, E2b-]-MUC1, or Tri-Ad5, respectively.

To determine the propensity of the CEA antibodies in the sera of Ad5 [E1-, E2b-]-CEA or Tri-Ad5 vaccinated mice to lyse tumor cells expressing CEA, we utilized a complement-dependent cytotoxicity (CDC) assay. Heat-inactivated sera from vaccinated mice were incubated with MC38-CEA2 tumor cells (murine CEA colon carcinoma cells transfected with human CEA), followed by rabbit sera as a source of complement. Lysis was determined by the release of lactate dehydrogenase (LDH) from MC38-CEA2 cells. There was significant lysis of MC38-CEA2 cells in sera from mice vaccinated with Tri-Ad5 or Ad5 [E1-, E2b-]-CEA, and this effect was similar between the two groups (Figure 4B).

**Figure 5:**
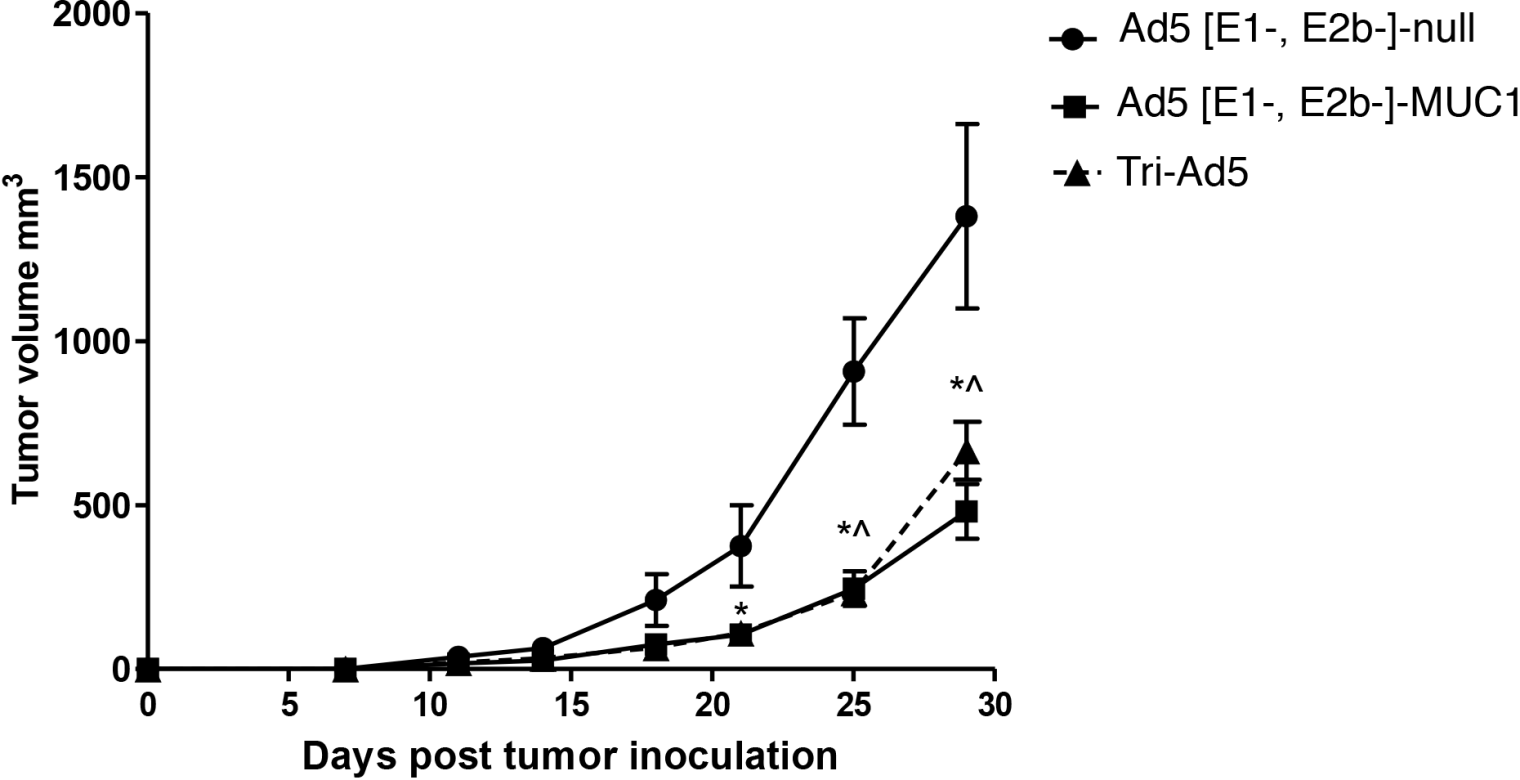
Comparison of immunotherapy of MUC1-expressing tumors using Ad5 [E1-, E2b-]-MUC1 vs. Tri-Ad5 C57Bl/6 mice (*n* = 7/group) were inoculated with 10^6^ MC-38-MUC1 cells subcutaneously in the left flank. Mice were administered 10^10^ VP (viral particle) of Ad5 [E1-, E2b-]-MUC1 or Tri-Ad5 (1:1:1 mixture of 10^10^ VP each of Ad5 [E1-, E2b-]-CEA, Ad5 [E1-, E2b-]-MUC1, and Ad5 [E1-, E2b-]-brachyury, 3 × 10^10^ VP total). A control group of mice received 3 × 10^10^ VP of Ad5 [E1-, E2b-]-null (no transgene). Tumor growth was monitored and volumes calculated. (*) indicates days when Ad5 [E1-, E2b-]-MUC1 treated mice had significantly smaller (*p* < 0.05) tumors than control mice and (^) indicates days when Tri-Ad5–treated mice had significantly smaller (*p* < 0.05) tumors than control mice. There was no significant difference (*p* > 0.1) between Ad5 [E1-, E2b-]-MUC1 vs. Tri-Ad5–treated mice at any time point. Error bars represent the SEM.

Studies were then undertaken to determine whether the Tri-Ad5 vaccine regimen was as effective as the use of a single recombinant adenovirus construct in eliciting an anti-tumor effect. C57BL/6 mice (*n* = 7/group) were implanted s.c. with 1 × 10^6^ MC38 cells expressing MUC1 (MC38-MUC1) in the left flank. Mice were vaccinated weekly with s.c. injections in the opposite flank using 10^10^ VP of Ad5 [E1-, E2b-]-MUC1 or Tri-Ad5, respectively. A control group of mice received 3 × 10^10^ VP of Ad5 [E1-, E2b-]-null (no transgene). Mice vaccinated with Ad5 [E1-, E2b-]-MUC1 or Tri-Ad5 had significantly smaller tumors than control mice on days 25 (*p* < 0.01) and 29 (*p* < 0.05) (Figure 5). There was no significant difference (*p* > 0.1) in anti-tumor effect for the groups of mice vaccinated with Ad5 [E1-, E2b-]-MUC1 or Tri-Ad5 at all time points.

## DISCUSSION

Recent clinical studies have brought cancer immunotherapy into the area of the management of several tumor types. The U.S. Food and Drug Administration (FDA) approval of the checkpoint inhibitor anti-CTLA4 and the Provenge prostate cancer vaccine has been followed by recent FDA approvals of anti-PD-L1/PD-1 immune checkpoint inhibitors [65–67]. The phenomenon of tumor heterogeneity, including diversity of TAA expression, is well established. The previously described [16, 22–25, 31, 32, 52–56, 68–71] wide level expression of CEA, brachyury, and MUC1 in a range of human carcinomas, along with their diverse activity in human tumors, renders the simultaneous targeting of these three TAAs of potential clinical benefit. We thus set out to determine whether a mixture of three recombinant adenovirus-TAA vaccines would be as effective as the use of each one individually.

The generation and use in a preclinical model of the recombinant Ad5 [E1-, E2b-]-CEA vaccine have been described previously [6]. A clinical trial of the Ad5 [E1-, E2b-]-CEA (ETBX-011) vaccine in patients with metastatic colorectal cancer demonstrated the ability to administer multiple vaccinations to immunocompromised patients safely, and provided a favorable survival profile [1, 10]. The potential advantages of targeting brachyury and MUC1 (including the C-terminus of MUC1) have also been described previously [24, 34, 72], as has the use of vaccines that also contain enhancer agonist epitopes of these TAAs [50, 51, 62–64].

In the studies reported here, we demonstrate that multi-TAA targeted immunotherapy (Tri-Ad5), which consists of a mixture of three Ad5 vectors expressing different TAAs, is as efficient in the activation of human T cells as the use of each of the adenovirus vectors alone, with only minor differences. We analyzed nine different *in vivo* parameters via vaccinating mice with each of the Ad5 [E1-, E2b-]-CEA, Ad5 [E1-, E2b-]-MUC1, and Ad5 [E1-, E2b-]-brachyury vectors individually versus vaccination with Tri-Ad5 (Figures 2–5). Of the 21 assays performed, the only statistical differences observed were (a) an enhanced number of MUC1-specific splenocytes and CD8^+^ IFN-producing and multifunctional CD8^+^ T cells, and (b) more CEA-specific CD8^+^ IFN-producing T cells, in the mice vaccinated with one vector than in the Tri-Ad5 vaccinated mice. On the other hand, the Tri-Ad5 vaccinated mice produced more CEA-specific IL-2–producing cells than the Ad5 [E1-, E2b-]-CEA vaccinated mice. In the other 16/21 assays, however, there were no statistical differences in the results between the use of the individual vector versus the use of the Tri-Ad5 in terms of antigen-specific activation of (a) splenocytes for IFN-γ and IL-2 production, (b) CD8^+^ T cells for IFN-γ production, (c) CD4^+^ T cells for IFN-γ production, (d) multifunctional CD8^+^ T cells for IFN-γ and TNF-α production, (e) multifunctional CD4^+^ T cells for IFN-γ and TNF-α production, and (f) production of antigen-specific antibodies (Figures 2–5). There was also no difference in anti-tumor activity using the single vector Ad5 [E1-, E2b-]- MUC1 versus the Tri-Ad5 vaccine; while both vaccines did not eliminate the tumor, both vaccines reduced the tumor growth rate in a similar manner. It should be noted that the reduction of tumor growth rate has been seen with several forms of immunotherapy in clinical studies [66, 73, 74]. It should also be pointed out that while Tri-Ad5 was not as efficient in T-cell activation in some assays, we believe that the potential ability of the Tri-Ad5 platform to overcome the TAA heterogeneity that exists in human solid tumors far outweighs the relatively minor differences in potency of T-cell activation of Tri-Ad5 vs. individual vectors in some assays.

CEA, MUC1 and brachyury are all human TAAs and are not expressed in murine solid tumors. Moreover, human solid tumors are very heterogeneous with respect to expression of different TAAs. It would be extremely difficult to transfect a murine tumor cell line with all three transgenes to define the effect of vaccination of Tri-Ad5 vs. each vector alone. We chose to use the targeting of a murine tumor expressing MUC1 because the single Ad5 [E1-, E2b-]-MUC1 vector was more potent in some of the murine T-cell assays (Figures 2 and 3) compared to Tri-Ad5 than the CEA and brachyury vectors compared to Tri-Ad5. Thus this appeared to be the most stringent model to compare the Tri-Ad5 platform to a single vector platform. The studies reported herein were designed to provide the rationale for potential clinical studies as a vaccine immunotherapy, or use in combination with other therapeutics, using this novel adenovirus vaccine delivery platform (Ad5 [E1-, E2b-]) targeting a diverse range of TAA transgenes in the Tri-Ad5 regimen.

Several other vaccine platforms are currently being evaluated in clinical studies that target CEA, MUC1, or brachyury. We have previously shown [75] in preclinical studies that two diverse vaccine platforms targeting the same TAA can and will induce quite different T-cell populations, including diverse epitope specificity, cytokine production, and avidity. It is thus speculated that different vaccine platforms targeting the same TAAs can be used in tandem in clinical studies to obtain a more diverse T-cell population, resulting in enhanced anti-tumor activity.

While the checkpoint inhibitor antibodies have shown evidence of clinical activity in melanoma and squamous non-small cell lung cancer, clinical benefit in other cancer types has been observed in a minority of patients. For some tumor types, such as colorectal cancer and prostate cancer, the anti-PD-L1/PD-1 checkpoint inhibitors have shown little clinical activity. One hypothesis that has been put forth for the lack of PD-L1/PD-1 therapeutic activity in some patients is the lack of T-cell infiltrates in tumors. Consequently, if a vaccine targeting TAAs in the tumor would result in the presence of antigen-specific T cells in the tumor microenvironment, then a checkpoint inhibitor employed in combination or following vaccination would be able to “release the brakes” of the tumor-infiltrating anergized T cells leading to clinical effect.

## MATERIALS AND METHODS

### Viral construction

Ad5 [E1-, E2b-]-brachyury, Ad5 [E1-, E2b-]- CEA and Ad5 [E1-, E2b-]-MUC1 were constructed and produced as previously described [6, 12]. Briefly, the transgenes were subcloned into the E1 region of the Ad5 [E1-, E2b-] vector using a homologous recombination-based approach. The replication deficient virus was propagated in the E.C7 packaging cell line, CsCl_2_ purified, and titered as previously described [12]. Viral infectious titer was determined as plaque-forming units (PFUs) on an E.C7 cell monolayer. The VP concentration was determined by sodium dodecyl sulfate (SDS) disruption and spectrophotometry at 260 nm and 280 nm (ViraQuest, North Liberty, IA). The CEA transgene also contains a modified CEA containing the highly immunogenic epitope CAP1–6D [63, 64].

The sequence encoding for the human brachyury protein (T, NM_003181.3) was modified by introducing the enhancer T-cell HLA-A2 epitope (WLLPGTSTV) [62] and removal of a 25 amino acid fragment involved in DNA binding. The resulting construct was subsequently subcloned into the Ad5 vector to generate the Ad5 [E1-, E2b-]-brachyury construct.

The MUC1 molecule consists of two regions: the N-terminus (MUC1-N), which is the large extracellular domain of MUC1, and the C-terminus (MUC1-C), which has three regions: a small extracellular domain, a single transmembrane domain, and a cytoplasmic tail [76]. The cytoplasmic tail contains sites for interaction with signaling proteins and has been shown to act as an oncogene and a driver of cancer motility, invasiveness and metastasis [56, 77]. For construction of the Ad5 [E1-, E2b-]-MUC1, the entire MUC1 transgene, including eight agonist epitopes previously described [50, 51], was subcloned into the Ad5 vector. The agonist epitopes included in the Ad5 [E1-, E2b-]-MUC1 vector bind to HLA-A2 (epitope P93L in the N-terminus, V1A and V2A in the VNTR region, and C1A, C2A and C3A in the C-terminus), HLA-A3 (epitope C5A), and HLA-A24 (epitope C6A in the C-terminus) [50, 51]. The Tri-Ad5 vaccine was produced by combining 10^10^ VP of Ad5 [E1-, E2b-]-brachyury, Ad5 [E1-, E2b-]-CEA and Ad5 [E1-, E2b-]-MUC1 at a ratio of 1:1:1 (3 × 10^10^ VP total). The vaccines used in this study are available to qualified researchers under a material transfer agreement.

### Generation of human DCs from PBMCs

Dendritic cells were generated from the peripheral blood mononuclear cells (PBMCs) of a prostate cancer patient (HLA-A2^+^ and -A24^+^) enrolled in a clinical trial employing a PSA-TRICOM vaccine in combination with ipilimumab [35], using the method previously described [78]; using PBMCs from this patient post-vaccination, we were able to establish individual T-cell lines specific for CEA, MUC1, and brachyury. An Institutional Review Board of the National Institutes of Health (NIH) Clinical Center approved the procedures, and informed consent was obtained in accordance with the Declaration of Helsinki. Briefly, PBMCs were isolated using lymphocyte separation medium gradient (ICN Biochemicals, Aurora, VA), resuspended in AIM-V medium (Invitrogen, Carlsbad, CA) (2 × 10^7^ cells) and allowed to adhere in a 6-well plate for 2 hours. Adherent cells were cultured for 5 days in AIM-V medium containing 100 ng/ml of recombinant human (rh) GM-CSF and 20 ng/ml of rhIL-4. The culture medium was replenished every 3 days.

### Infection of human DCs with adenovirus vectors

Dendritic cells (2 × 10^5^) in 1 ml of AIM-V medium were infected with adenovirus vectors (Ad5 [E1-, E2b-]-CEA, Ad5 [E1-, E2b-]-MUC1, Ad5 [E1-, E2b-]-brachyury, and Ad5 [E1-, E2b-]-null at indicated multiplicity of infection (MOI of 10,000 or 20,000) for 1 hour in 6-well plates. AIM-V medium (4 ml) was then added to each well and incubated for an additional 2 days. To analyze the efficacy of transgene expression, DCs were harvested and analyzed using flow cytometry and Western blot. For phenotypic analysis, DCs were stained for the expression of CD80, CD83, CD86, CEA, and HLA-DR using BV421-conjugated anti-CD80, PerCP Cy5.5-conjugated anti-CD83, APC-Cy7-conjugated anti-HLA-DR, PE-conjugated anti-CD86, and FITC-conjugated anti-CEA. Antibodies for flow cytometry were purchased from BD Bioscience (San Jose, CA).

### Generation of T-cell lines using adenovirus-infected DCs

A modification of the method described by Tsang et al. [79] was used to generate CEA-, MUC1- and brachyury-specific cytotoxic T lymphocytes (CTLs). Dendritic cells (1–2 × 10^5^ /well in 1 ml of AIM-V) were infected with 20,000 MOI of Tri-Ad5, as described above. Infected DCs were used as APCs for stimulation of autologous nonadherent cells at an effector-APC ratio of 10:1. Cultures were incubated for 3 days at 37°C in a humidified atmosphere containing 5% CO_2_. The cultures were then supplemented with rhIL-2 for 7 days; IL-2 containing medium was replenished every 3 days. The 10-day stimulation constituted one *in vitro* stimulation (IVS) cycle. Autologous vector-infected DCs were used as APCs for three IVS. Autologous peptide-pulsed B cells were used to restimulate antigen-specific CTLs after three IVS. T-cell lines were maintained in medium containing IL-7 and IL-15 (10 ng/ml; PeproTech, Rocky Hill, NJ).

### Cytotoxic assay

A modification of the protocol described by Tsang et al. [80] was used for CTL analysis. In brief, target cells were labeled with 50 μCi of ^111^In oxide (GE Health Care, Vienna, VA) at 37°C for 20 min and used at 3,000 cells/well in 96-well round-bottom culture plates. T cells were added at different ratios and incubated at 37°C for 16 hours. Supernatants were harvested for gamma counting. Determinations were carried out in triplicate and SDs were calculated. Spontaneous release was determined by incubating target cells with medium alone and complete lysis was determined by incubating with 0.25% Triton X-100. Specific lysis was calculated with the use of the following formula: Lysis (%) = [observed release (CPM)- spontaneous release (CPM)] / [Complete release (CPM)- spontaneous release (CPM)] × 100.

### Tumor cell culture

Human colon carcinoma SW620 (HLA-A2^+^, HLA-A24^+^, brachyury^+^, MUC1^+^, CEA^+^) and pancreatic carcinoma ASPC-1 (HLA-A1^+^, HLA-A26^+^, MUC1^+^, brachyury+, CEA+) cell lines were obtained from American Type Culture Collection (Manassas, VA). Cell cultures were free of mycoplasma and maintained in complete medium (RPMI-1640 supplemented with 10% FBS, 100 U/ml penicillin, 100 μg/ml streptomycin and 2 mM L-glutamine) (Mediatech, Herndon, VA).

### Detection of cytokines

Supernatants of T cells stimulated for 24 hours with DCs infected with adenovirus vectors or peptide-pulsed DCs in IL-2–free medium were evaluated for secretion of IFN-γ using an ELISA kit (Invitrogen, Frederick, MD). The antigen-specific T-cell lines used in this analysis have been reported previously: (a) an HLA-A2 CEA-specific CTL [81], (b) an HLA-A2 MUC1-specific CTL [50], (c) an HLA-A24 MUC1-specific CTL [51], and (d) an HLA-A2 brachyury-specific CTL [62].

### Peptides

The following HLA-A2 and HLA-A24 binding peptides were used in this study: (a) the HLA-A2 binding CEA agonist peptide CAP1–6D (YLSGADLNL) [64], (b) the HLA-A2 MUC1 agonist peptide P93L (ALWGQDVTSV) [50], (c) the HLA-A24 binding MUC1 agonist peptide C6A (KYHPMSEYAL) [51], and (d) the HLA-A2 binding brachyury agonist peptide (WLLPGTSTV) [62]. All peptides were greater than 96% pure and manufactured by American Peptide Company, Inc. (Sunnyvale, CA).

### Mice

Specific pathogen-free, female C57BL/6 mice (Jackson Laboratory, Bar Harbor, ME) of ages 8−10 weeks were housed in animal facilities at the Infectious Disease Research Institute (IDRI) (Seattle, WA, USA). All procedures were conducted according to Institutional Animal Care and Usage Committee (IACUC) approved protocols.

### Vaccination and splenocyte preparation

Female C57BL/6 mice (*n* = 5) were injected s.c. with 10^10^ VP of Ad5 [E1-, E2b-]-brachyury or Ad5 [E1-, E2b-]-CEA or Ad5 [E1-, E2b-]-MUC1 or a combination of 10^10^ VP of all three viruses at a ratio of 1:1:1 (Tri-Ad5). Control mice were injected with 3 × 10^10^ VP of Ad5 [E1-, E2b-]-null (no transgene insert). Doses were administered in 25 μ1 of injection buffer (20 mM HEPES with 3% sucrose) and mice were vaccinated three times at 14-day intervals. Fourteen days after the final injection spleens and sera were collected. Sera were frozen at −20°C. Splenocyte suspensions were generated by gently crushing the spleens through a 70 μM nylon cell strainer (BD Falcon, San Jose, CA). Red cells were removed by the addition of red cell lysis buffer (Sigma-Aldrich, St. Louis, MO) and the splenocytes were washed twice and resuspended in R10 (RPMI 1640 supplemented with L-glutamine (2 mM), HEPES (20 mM) (Corning, Corning, NY), penicillin 100 U/ml and streptomycin 100 μg/ml (Hyclone, GE Healthcare Life Sciences, Logan, UT), and 10% fetal bovine serum (Hyclone). Splenocytes were assayed for cytokine production by ELISPOT and flow cytometry.

### ELISPOT assay

Brachyury-, CEA- and MUC1-specific IFN-γ– or IL-2–secreting T cells were determined by ELISPOT assay from freshly isolated mouse splenocytes, as described above. The ELISPOT assay was performed according to the manufacturer's specifications (Affymetrix Bioscience, San Diego, CA). Briefly, 2 × 10^5^ splenocytes were stimulated with 0.2 μg/well of overlapping 15-mer peptides in a single pool derived from brachyury or CEA (JPT Peptide Technologies, Berlin, Germany) or MUC1. Cells were stimulated with Concanavalin A (Con A) at a concentration of 0.0625 μg/per well as a positive control and overlapping 15-mer complete peptides pools derived from SIV-Nef and SIV-Vif (AIDS Research and Reference Reagent Program, Division of AIDS, National Institute of Allergy and Infectious Diseases (NIAID), National Institutes of Health (NIH)) were used as irrelevant peptide controls. The numbers of SFCs were determined using an Immunospot ELISPOT plate reader (Cellular Technology, Shaker Heights, OH) and results were reported as the number of SFCs per 10^6^ splenocytes.

### Intracellular cytokine stimulation

Splenocytes were prepared as indicated above. Stimulation assays were performed using 1 × 10^6^ live splenocytes per well in 96-well U-bottom plates. Pools of overlapping peptides spanning the entire coding sequences of brachyury, CEA and MUC1 were synthesized as 15-mers with 11-amino acid overlaps (JPT GmbH) and lyophilized peptide pools were dissolved in Dimethyl sulfoxide (DMSO). Similarly constructed peptide pools corresponding to SIV-Vif and SIV-Nef served as off-target controls. Splenocytes in R10 media (RPMI 1640, 10% fetal bovine serum, and antibiotics) were stimulated by the addition of peptide pools at 2 μg/mL/peptide for 6 h at 37°C and 5% CO_2_, with protein transport inhibitor (GolgiStop, BD) added 2 hours into the incubation. Stimulated splenocytes were then stained for lymphocyte surface markers CD8α and CD4, fixed, permeabilized, and then stained for the intracellular accumulation of IFN-γ and TNF-α. Antibodies against mouse CD8α (clone 53–6.7), CD4 (clone RM4–5), IFN-γ (clone XMG1.2), and TNF-α (clone MP6-XT22) were purchased from BD and staining was performed in the presence of anti-CD16/CD32 (clone 2.4G2). Flow cytometry was performed using an Accuri C6 Flow Cytometer (BD) and analyzed in BD Accuri C6 Software.

### ELISA to detect antibodies against CEA

ELISA plates (Nunc Maxisorp, Sigma-Aldrich, st Louis, mo) were coated with 100 ng of human CEA in 0.05M carbonate-bicarbonate buffer pH 9.6 and incubated overnight at room temperature. Plates were washed three times with phosphate buffered saline containing 1% Tween-20 (PBS-T) and then blocked with PBS containing 1% BSA for 60 min at room temperature. After an additional three washes, sera diluted 1/50 in PBS-T were added to the wells and the plates were incubated for 1 hour at room temperature. Peroxidase labeled goat anti-mouse immunoglobulin (Ig) G (γ-chain specific) (Sigma-Aldrich) antibody at a 1:5000 dilution was added to the wells after washings and plates were incubated for 1 hour. Plates were washed three times and 1,2-phenylene-diamine substrate solution (Thermo-Fisher Scientific, Waltham, MA) was added to each well. The reaction was stopped by adding 10% phosphoric acid. Absorbance was measured at 492 nm on a SpectraMax 190 ELISA reader (Molecular Devices, Sunnyvale, CA). The nanogram equivalents of IgG bound to CEA per well were obtained by reference to a standard curve generated using purified mouse IgG and developed at the same time as the CEA ELISA (Sigma-Aldrich) as previously described [5]. The results were analyzed and quantitated using SoftMax Pro 6.3 software (Molecular Devices).

### Complement-dependent cytotoxicity assay (CDC)

MC38-CEA2 tumor cells were cultured overnight at a density of 2 × 10^4^ cells per well in 96-well tissue culture microplates. Pooled heat inactivated mouse sera were added at a 1:50 dilution and incubated at 37°C for 1 hour. Rabbit serum was then added at a 1:50 dilution as a source of complement and cells were incubated an additional 2.5 hours at 37°C. Cell culture supernatants were assayed using Promega Cytotox 96 non-radioactive cytotoxicity assay (Promega, Madison, WI), according to the manufacturer's instructions. Percent lysis of MC38-CEA2 cells was calculated by the formula % lysis = (experimental – target spontaneous) / (target maximum – target spontaneous) × 100%.

### Tumor immunotherapy

For *in vivo* tumor treatment studies, female C57BL/6 mice, 8–10 weeks old, were implanted with 10^6^ MC38-MUC1 cells s.c. in the left flank. Mice were treated three times at a 7-day interval with 10^10^ VP Ad5 [E1-, E2b-]-MUC1 or Tri-Ad5. Control mice were injected with 3 × 10^10^ VP of Ad5 [E1-, E2b-]-null. Tumor growth was assessed by measuring two opposing dimensions (*a, b*) and the volume calculated as previously described [82] according to the formula *V* = (axb)^2^/2 where the shorter dimension was “*a*”. Tumor studies were terminated when tumors reached 1500 m^3^ or became severely ulcerated.

### Supplemental materials

Supplemental data for this manuscript are available online at the publisher's website.

## SUPPLEMENTARY DATA



## Abbreviations

Ad5: Adenovirus serotype-5
Ad5 [E1-]: Adenovirus serotype-5 (Ad5)-based vector platforms with deletions in the early 1 (E1) gene and early 3 (E3) gene regions
Ad5 [E1-, E2b-]: Ad5 [E1-] with additional deletions in the early 2 (E2) gene region and early (E3) gene region
APC: antigen presenting cell
CEA: carcinoembryonic antigen
CDC: complement-dependent cytotoxicity
Con A: Concanavalin A
CTL: cytotoxic T lymphocyte
DC: dendritic cell
DMSO: dimethyl sulfoxide
ELISA: enzyme-linked immunosorbent assay
ELISPOT: enzyme-linked immunospot
EMT: epithelial-mesenchymal transition
FDA: U.S. Food and Drug Administration
GM-CSF: granulocyte-macrophage colony-stimulating factor
HIV: human immunodeficiency virus
IFN: interferon
Ig: immunoglobulin
IVS: in vitro stimulation
LDH: lactate dehydrogenase
MAb: monoclonal antibody
mCRC: metastatic colorectal cancer
MHC: major histocompatibility complex
MOI: multiplicity of infection
MUC1: cancer-associated mucin
PBMC: peripheral blood mononuclear cells
PFU: plaque-forming unit
PSA: prostate-specific antigen
s.c.: subcutaneously
SDS: sodium dodecyl sulfate
SFC: spot forming cells
SIV: simian immunodeficiency virus
TAA: tumor-associated antigen
Tri-Ad5: recombinant adenovirus admixture consisting of Ad5 [E1-, E2b-]-CEA, Ad5 [E1-, E2b-]-brachyury and Ad5 [E1-, E2b-]-MUC1
VP: viral particles
