# Supplementary material for: The generation and analyses of a novel combination of recombinant adenovirus vaccines targeting three tumor antigens as an immunotherapeutic
Source: Oncotarget. 2015 Sep 7;6(31):31344–59. doi: 10.18632/oncotarget.5181 (PMC4741610; doi:10.18632/oncotarget.5181)
Supplement: Supplementary file 1 [file oncotarget-06-31344-s001.pdf]

SUPPLEMENTARY DATA

METHODS

A549 cells were inoculated at an MOI of 555 VP/cell with Ad5 [E1-, E2b-]-CEA. Cells were incubated for 48 hours at 37°C in 5% CO_2. After 48 hours cells were harvested and washed with PBS and freeze/thawed three times. The whole cell lysate was heated at 70°C for 10 minutes prior to loading on the gel. Recombinant CEA control was loaded at 30 ng/lane and the prepared lysate at 20 uL/lane. Sample loading buffer was included as an additional negative control and the positive controls were Magic Mark CP Western Markers and the recombinant CEA. The gel was transferred to a nitrocellulose membrane and blocked with SuperBlock Blocking solution for 60 mins. The membrane was probed with mouse monoclonal anti-CEA primary antibody (1:1000) and a secondary anti-mouse HRP (1:2500) conjugated antibody. The membrane was washed three times then incubated with SuperSignal chemiluminescent reagent and banding was visualized by exposing X-ray film to the membrane followed by development.

Supplementary Table S1: Serotype 5 adenovirus (Ad5) vectors induce maturation and activation of human DCs

Treatment	MOI	CD80	CD83	CD86	HLA-DR
Control	0	20.2 (146)	33.6 (259)	99.5 (4586)	94.4 (1355)
Ad5 [E1-, E2b-]-null	10,000	**40.7 (162)**	**40.9 (253)**	99.2 (3794)	**98.0 (4489)**
Ad5 [E1-, E2b-]-null	20,000	**47.4 (169)**	**46.8 (266)**	98.6 (3012)	**95.7 (3203)**
Ad5 [E1-, E2b-]–CEA	10,000	**56.8 (189)**	**48.7 (262)**	99.2 (3877)	**98.3 (6553)**
Ad5 [E1-, E2b-]–CEA	20,000	**54.5 (185)**	**46.6 (271)**	99.1 (3628)	**98.0 (6015)**
Ad5 [E1-, E2b-]–MUC1	10,000	**41.4 (167)**	**42.4 (251)**	98.5 (3178)	**96.9 (5227)**
Ad5 [E1-, E2b-]–MUC1	20,000	**46.7 (172)**	**44.3 (260)**	98.9 (3591)	**97.2 (5779)**

Indicated adenovirus vectors were used at a concentration of 10,000 or 20,000 multiplicity of infection (MOI) for infection of human dendritic cells (DCs). Expression of CD80, CD83, CD86 and HLA-DR were analyzed by flow cytometry. Results are expressed in % of positive cells (mean fluorescence intensity).

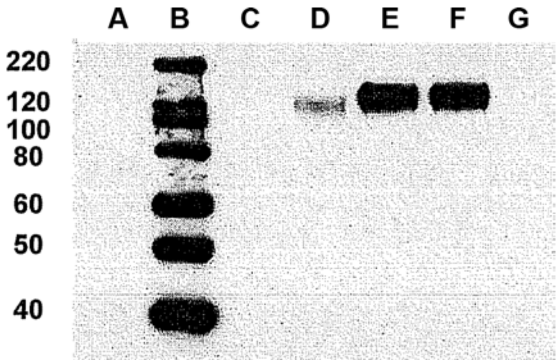

Supplementary Figure S1: Expression of CEA in A549 cells infected with Ad5 [E1-, E2b-]-CEA. A549 cells were infected with Ad5 [E1-, E2b-]-CEA and CEA expression was confirmed by Western blot analysis. Recombinant CEA was used as a positive control and uninfected A549 cells served as a negative control. The samples are visualized above in the following order: A. Negative Control, B. Magic Mark XP Western Marker, C. Negative, D. CEA Reference Material (30 ng), E. Ad5 [E1-, E2b-]-CEA lysate (20uL), F. Ad5 [E1-, E2b-]-CEA lysate (20 uL), G. Negative A549 cells.
